# Chromium carbide/Carbon Nanotube Hybrid Structure Assisted Copper Composites with Low Temperature Coefficient of Resistance

**DOI:** 10.1038/s41598-017-14915-7

**Published:** 2017-11-02

**Authors:** Seungchan Cho, Keiko Kikuchi, Eunkyung Lee, Moonhee Choi, Ilguk Jo, Sang-Bok Lee, Sang-Kwan Lee, Akira Kawasaki

**Affiliations:** 1 Composites Research Division, Korea Institute of Materials Science (KIMS), Changwon, 51508 South Korea; 2 Department of Materials Processing, Graduate School of Engineering, Tohoku University, Sendai, 980-8579 Japan; 3 Mechanical Engineering, Worcester Polytechnic Institute, Worcester, MA01609 USA; 4 Samsung Electro-Mechanics, Suwon, 16674 South Korea

## Abstract

In order to explore the possibility of using carbon nanotube (CNT) to introduce and control the temperature coefficient of resistance (TCR) of metal matrix composite, relatively thick and short multi-walled CNTs (MWCNTs) were introduced in the metal matrix with *in-situ* formation of chromium carbide (Cr_7_C_3_) at the CNT/copper (Cu) interface. We demonstrate that incompatible properties such as electrical conductivity and TCR can be achieved simultaneously by introducing MWCNTs in the Cu matrix, with control of the interfacial resistivity using the MWCNT/Cr_7_C_3_–Cu system. High electrical conductivity of 94.66 IACS and low TCR of 1,451 10^–6^ °C^−1^ are achieved in the 5 vol.% MWCNT–CuCr composite. *In-situ* formation of Cr_7_C_3_ nanostructures at the MWCNT/Cu interface by reaction of diffused Cr atoms and amorphous carbon of MWCNTs would assist in improving the electrical properties of the MWCNT–CuCr composites.

## Introduction

With increasing demand for faster, smaller electronic devices in ultralarge-scale integrated devices (ULSI), copper (Cu), with a low resistivity and a superior electromigration resistance, is a promising conductor material compared to conventional aluminum (Al)^1,2^. However, the high temperature coefficient of resistance (TCR) of about 4,000 ppm °C^−1^ of pure Cu is an obstacle for precise conductivity control of a device due to the heat generated during operation. Resistors are the most commonly used passive component in electronics; their purpose is to create specified values of current and voltage in a circuit. Currently, metal resistors, as current sensing devices in electronics and automotive applications, have attracted great attention owing to their ultra-low resistance (0.5~10mΩ) and precise value of resistance (±0.5%) with high power. TCR is one of the main parameters that must be controlled to fabricate low–ohm (Ω) resistors without temperature compensation. Therefore, the development of Cu matrix composites having high electrical conductivity and low TCR will play a key role in electronic applications.

Tailoring of the physical and mechanical properties in a metal matrix by incorporating nano carbon materials such as carbon nanotube (CNT), graphene, carbon nanofiber has been a deeply studied topic in the recent years^3–11^. Since the discovery of carbon nanotubes (CNTs), which consist of rolled-up graphene sheets built from sp^2^ carbon units, there has been great interest in advanced composite materials due to their remarkable mechanical, electrical and thermal properties^12–15^. Especially, the addition of CNTs is expected to bring remarkable enhancement of the electrical and thermal conductivity in metals such as Cu or Al because of the extremely low electrical resistivity of multi-walled CNT (MWCNT) (reported to be as high as 3 × 10^–5^ Ωcm)^16,17^, indicating that CNTs may be better conductors than metals such as copper at room temperature. The electrical and thermal conductivities of such composites can be tailored by appropriate selection of CNT chirality, doping, crystallinity, and structure. The conductivities of individual CNTs are often much lower than defect-free CNTs under ballistic conduction^18–20^. Especially, the calculated TCR of CNT can be diverse according to the structural characteristics such as length, diameter, and level of impurity^21^. Furthermore, interfacial structure control plays a fundamental role in the design of materials for specific applications. Unlike traditional micrometer sized reinforcements, nanoscale fillers such as graphene and CNT have higher specific surface areas, and as such play crucial roles in determining the electrical properties of the composites because of the greatly increased interface area between the matrix and the CNTs. Successful tailoring of the material system in MMCs, however, has been rare due to the difficulty of improving what are generally thought to be mutually exclusive properties.

One of the most effective methods for decreasing the interfacial resistivity between the CNT and the metal matrix with low TCR is local carbide formation, which can aid electron–phonon coupling at the interface between the CNTs and the Cu matrix by adding a small amount of a carbide-forming element in the metal matrix because Cu does not react with carbon. We previously showed that chromium (Cr) carbide nanostructures generated in an MWCNT–Cu composite can increase the interfacial bonding strength of MWCNT/Cu without deteriorating the thermal conductivity of the composite^22^. Alloying the metal matrix with elements such as Cr, Zr, and Ti offers a route to improve the interfacial thermal conductivity in the diamond–Cu composite^23–26^.

Here, to lower the electrical resistivity and the TCR of the Cu composite via *in-situ* reaction during the spark plasma sintering (SPS) process, we have fabricated Cr carbide nanostructures on thick MWCNTs in an MWCNT–Cu composite; this method is effective for interfacial reaction control due to the relatively short sintering time at low temperature by joule heating, with various vol. % of MWCNTs. Resistivity and TCR of the fabricated MWCNT–Cu composite are evaluated and compared with those characteristics of conventional materials.

## Results and Discussions

### Structural characterization

**Figure 1:**
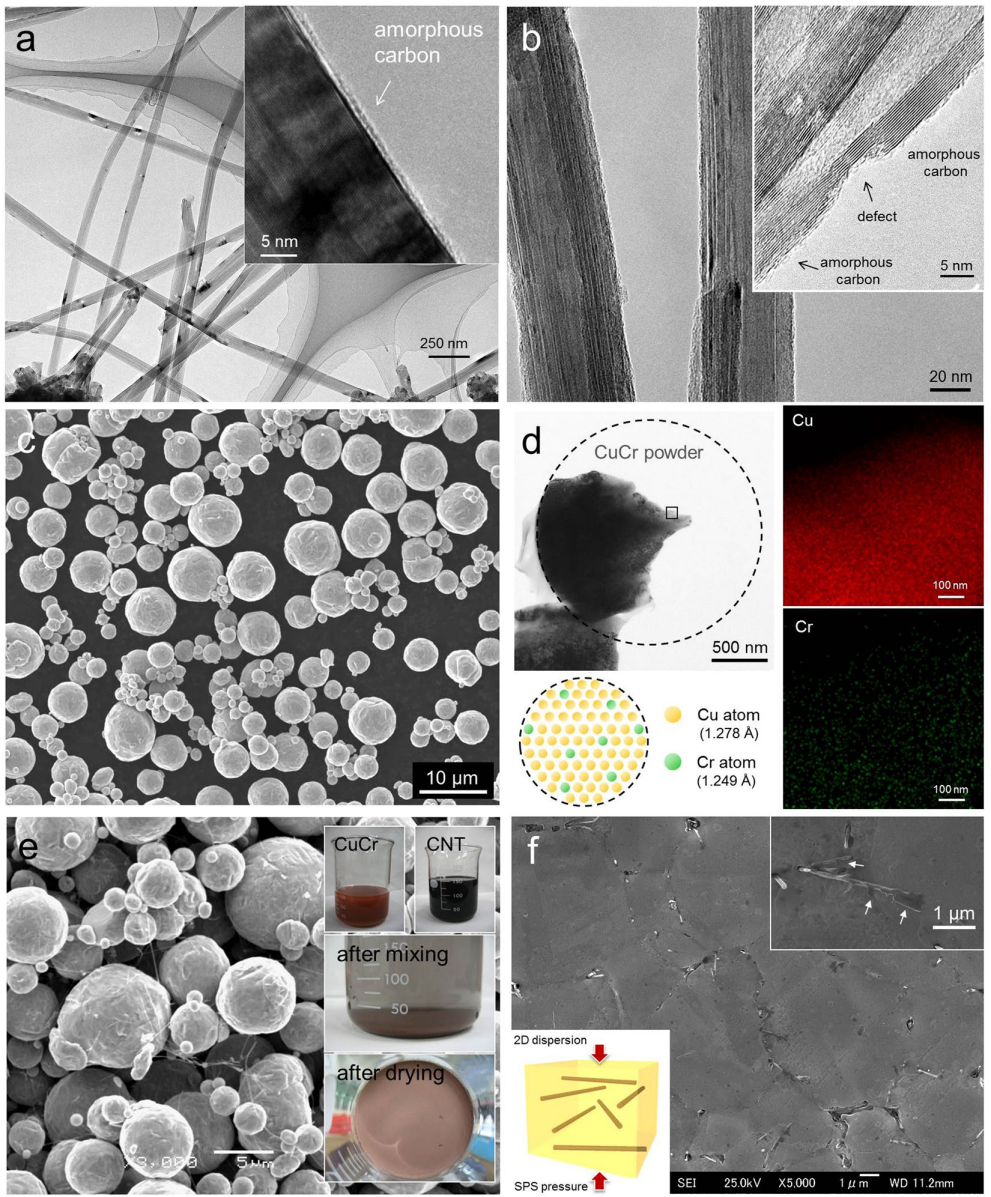
TEM images of (**a**) pristine MWCNTs and (**b**) chemically treated MWCNTs. (**c**) SEM and (**d**) TEM images with EDS element mapping of CuCr powder. (**e**) SEM image of MWCNT/CuCr mixture. Insets show photographs of wet mixing process. (**f**) FESEM image of sintered 3 vol.% MWCNT–CuCr composite. Insets show magnified SEM images of MWCNTs having precipitates on the surface and schematic of 2D distribution of CNTs in a plane normal to the SPS compression axis.

The pristine MWCNTs, with diameters ranging from 20–110 nm and length ranging from 2–15 μm, synthesized by means of chemical vapor deposition followed by high-temperature annealing at 2600 °C ^27^, were used in this research (Fig. 1). The MWCNTs were suspended in a mixture of concentrated H_2_SO_4_ and HNO_3_ with volume ratio of 3:1 and ultrasonicated in a water bath at 50 °C for 24 h. Figure 1b shows the chemically treated MWCNTs, which have partially amorphous carbon at the side surface of the MWCNTs, as shown in the inset of Fig. 1a. CuCr (0.085 at.% Cr) solid solution powder (Fukuda Metal Foil & Powder Co. LTD., Japan) produced by gas atomization under an inert Ar atmosphere was used in this study (Fig. 1c). The mean particle size of the CuCr alloy powder, determined using a particle size analyzer, was approximately 5 µm. Figure 1d shows a TEM image and energy dispersive spectroscopy (EDS) element mapping of the CuCr powders. It can be seen that there is no precipitation of Cr at the center of the powder, which has maintained a solid solution state owing to the extremely high cooling rate of the gas atomization process. Figure 1e provides an SEM image of the MWCNT–CuCr composite powder, showing homogeneously dispersed MWCNTs in the CuCr powder. The composite powder was prepared by surfactant-free process^7,8^. This process is simply based on the charge difference between the positively charged Cu particles and the MWCNTs, which are negatively charged by acid treatment in the solvent. The two solutions containing CuCr powder and MWCNTs were blended to generate a uniform mixture. The MWCNT–CuCr mixture sank to the bottom of the beaker in ethanol solution due to an attractive force between the negatively charged MWCNTs and the positively charged CuCr powders (Insets of Fig. 1e, S1). Figure 1f shows the microstructure of the 3 vol.% MWCNT–CuCr composites sintered at 800 °C for 1 min. The MWCNTs are relatively well dispersed at the grain boundaries throughout the matrix up to 5 vol.% MWCNT content (Fig. S2). The magnified FESEM image in the inset of Fig. 1f clearly shows that some of the MWCNTs (white arrow) appeared to react with the CuCr matrix; a shape change of the MWCNTs was observed, indicating the possibility of carbide formation. Furthermore, the CNTs were randomly oriented in a plane normal to the SPS compression axis, as illustrated in inset of Fig. 1f. Measured thermal conductivities and TEM images of the MWCNT–Cu composites fabricated by SPS process confirm 2D distribution of the MWCNTs in a plane normal to the SPS compression axis, which is effective at achieving in-plain conductivity of the composites, compared with random distribution due to their anisotropic conductivity (Fig. S3).

**Figure 2:**
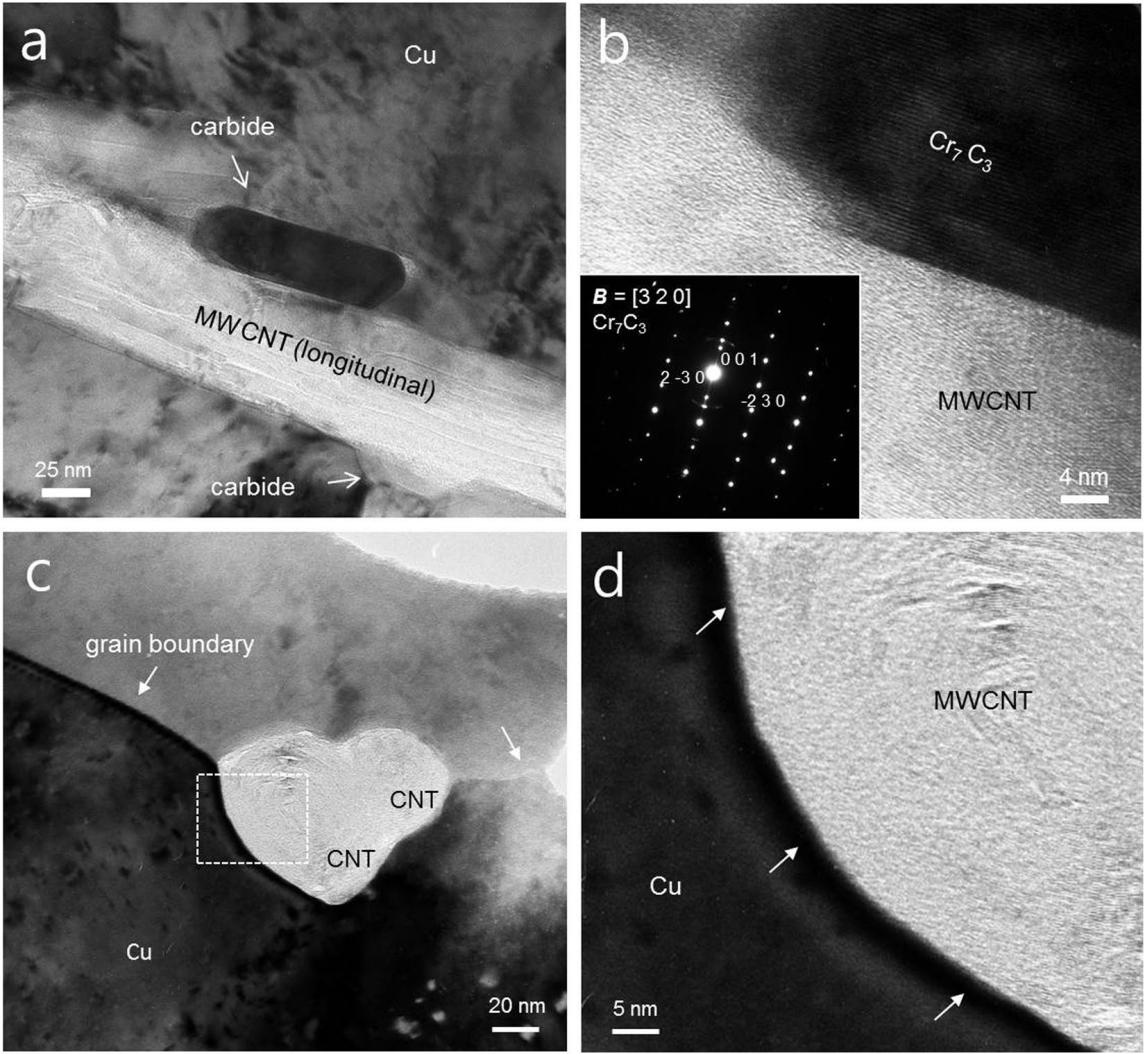
(**a**) TEM and (**b**) magnified TEM images of MWCNT/Cr carbide (Cr_7_C_3_) hybrid structure in the Cu matrix with electron beam diffraction pattern of Cr carbide. (**c**,**d**) Cross sectional TEM image of MWCNT/Cu interface.

Figure 2a provides a TEM image of a MWCNT/carbide hybrid structure in the CuCr matrix sintered at 990 °C for 10 min. The morphology is island-shaped, with a size of a hundred nanometers. The carbide nanostructure formed a plane parallel to the sidewall surface of the MWCNT, indicating epitaxial growth^22^. Thus, it is obvious that the Cr carbide nanostructure was generated by epitaxial growth to the outer direction from the side wall of the MWCNTs. The electron beam diffraction pattern obtained in nano-beam diffraction mode of the TEM clearly reveals that the nanostructure is single crystal Cr_7_C_3_ phase, as shown in the inset of Fig. 2b. Crystal structure of various Cr carbide nanostructures having a length of 100 nm or less observed in the MWCNT–CuCr composites was Cr_7_C_3_ phase as shown in Fig. S4.

Al_4_C_3_ formation is usually reported at the sides and tips of CNTs in the CNT–Al composite. However, these tendencies of carbide formation are different from the results in this study. In the Al–CNT composites, graphene layers of CNT changed to Al_4_C_3_ by reaction with diffused Al atoms, resulting in the formation of tube shaped Al_4_C_3_^28^. However, Cr_7_C_3_ nanostructures were grown mainly in a direction perpendicular to the outermost graphene layer of the MWCNTs. This difference is thought to have originated from a reactivity difference between the Al/CNT and the CuCr/CNT, because of the small amount of Cr content in the CuCr powder. Because chemically treated MWCNTs still include a small amount of amorphous carbon, which is the most reactive phase of carbon, the formation of carbide nanoparticles is expected to originate from a defective outer graphene layer and/or from amorphous carbon. MWCNT/Cr_7_C_3_ hybrid structure was observed at the fracture surface after tensile test of MWCNT–CuCr composite (Fig. S5). Most of the MWCNTs protrude beyond the fractured surface by a length of few hundred nanometers. The fractured microstructure after the tensile test confirmed that the carbide was attached to the MWCNT surface, indicating strong interfacial bonding strength between MWCNT and Cr_7_C_3_.

Because it is difficult to conduct precise observation of the MWCNT/Cu interface due to an overlap of graphene layers in the MWCNTs, a cross section of the sample was analyzed using TEM. Figure 2c shows a cross sectional TEM image of the 3 vol.% MWCNT–CuCr composite. There are no impurities or second phases along the grain boundaries or along the MWCNT/Cu interface of the sample. The high magnification TEM image of the MWCNT/Cu interface provided in Fig. 2d clearly shows an extremely clean interface. In our previous research, interfacial amorphous layers, thought to originate from Cu oxide in Cu powders and amorphous carbon on the surface of the MWCNTs, were observed in the MWCNT–Cu composites^7^. Thus, amorphous carbon may be selectively removed from the CNT surface in the MWCNT–CuCr composite.

**Figure 3:**
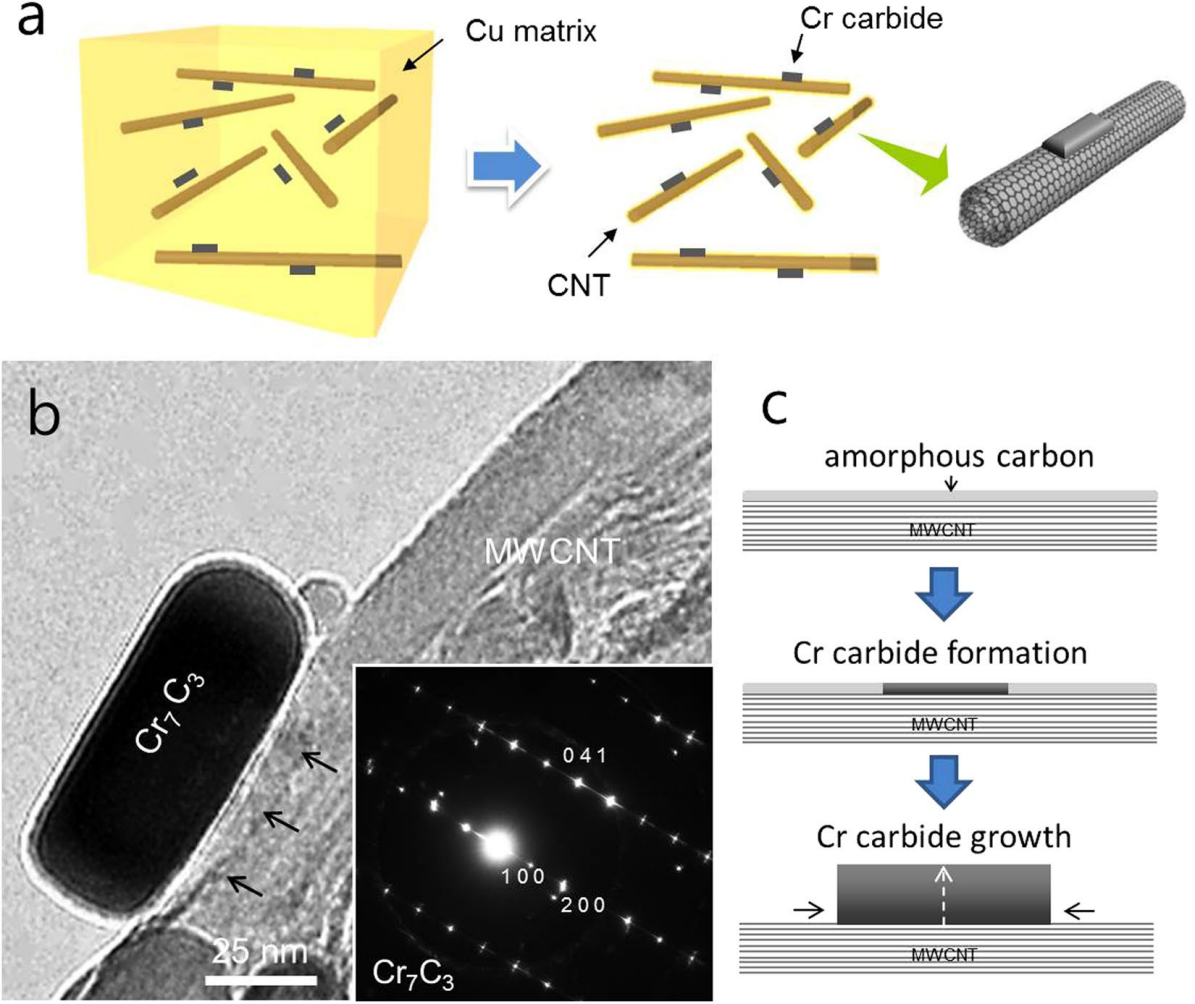
(**a**) Schematic of removing Cu matrix using 20 vol.% H_2_SO_4_ solution. (**b**) TEM image of Cr carbide nanostructure grown on MWCNTs observed after removing Cu matrix using 20 vol.% H_2_SO_4_ solution. (**c**) Possible schematic of carbide growth on MWCNTs by reaction of diffused Cr atoms and amorphous carbons on MWCNT.

In order to conduct a precise investigation of the MWCNT/carbide interface, the Cu matrix is removed using 20 vol.% H_2_SO_4_ solution, as shown in Fig. 3a; this removal can be expressed by the following equations.

1 $${\rm{Cu}}(s)+{{\rm{H}}}_{2}{\rm{O}}(l)\to {\rm{CuO}}(s)+{{\rm{H}}}_{2}(g)$$

2 $${\rm{CuO}}(s)+{{\rm{H}}}_{2}{{\rm{SO}}}_{4}(l)\to {{\rm{CuSO}}}_{4}(l)+{{\rm{H}}}_{2}{\rm{O}}(l)$$

There was no difference in the *I*_*D*_ */I*_*G*_ ratio of the MWCNTs before and after removing the Cu matrix, because the sample was dipped and resolved in mild acidic solution containing 20 vol.% H_2_SO_4_, indicating negligible structural damage of the MWCNTs. The magnified TEM image in Fig. 3b provides insight into the formation mechanism of the Cr carbide nanostructure in the MWCNT–CuCr composite. The TEM image clearly shows that the outermost graphene layer maintained its graphitic structure at the MWCNT/carbide interface, indicating no reaction between the MWCNTs and the Cr atoms. Therefore, this observation provides further evidence that the nanostructures were epitaxially grown on the side wall of the MWCNTs through the diffusion of solute Cr atoms to amorphous carbons attached on the MWCNTs, indicating *in-situ* removal of amorphous carbon impurities of the MWCNTs in the composite during the sintering process (Fig. 3c).

### Electrical characterization

**Figure 4:**
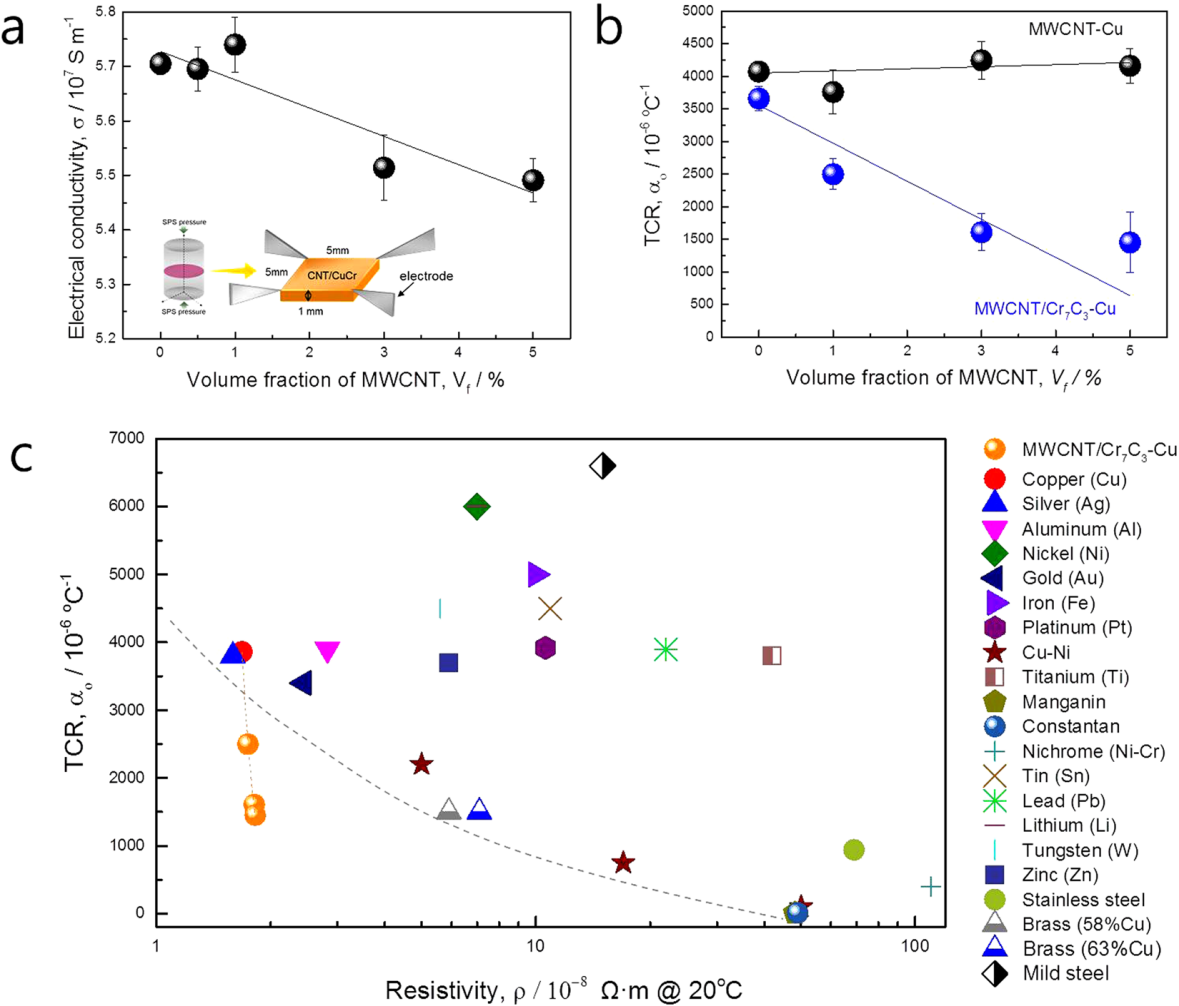
(**a**) Electrical conductivities and (**b**) TCRs of the MWCNT–CuCr composites as a function of MWCNT volume fraction. (**c**) TCR and electrical resistivity of the various materials^29,30^.

Figure 4a shows electrical conductivities and TCRs of the MWCNT–CuCr composites as a function of MWCNT volume fraction. Electrical conductivities of MWCNT–Cu composites slightly decreased with increasing MWCNT volume fraction, even though the electrical conductivities retain similar values for sintered CuCr up to 1 vol.% MWCNT. However, the 5 vol.% MWCNT–CuCr composite with electrical conductivity of 5.491 × 10^7^ S m^−1^ corresponds to 94.66% of the international annealed copper standard (IACS), as shown in Table 1.

**Table 1:** Densities and electrical properties of MWCNT–CuCr composites as a function of MWCNT content.

MWCNT vol.%	Density (g/cm^3^)	TCR @ 25–125 °C (10^−6^ °C^−1^)	Electrical Conductivity @RT (10^7^ S m^−1^)	IACS (%)
0	8.947 (RD 99.9%)	3658.2	5.705	98.36
1	8.841 (RD 99.5%)	2498.7	5.740	98.96
3	8.745 (RD 99.0%)	1608.3	5.514	95.07
5	8.562 (RD 99.5%)	1451.1	5.491	94.66

Figure 4b shows the TCR values of the MWCNT–CuCr composites and the MWCNT–Cu composites as a function of the MWCNT contents. While the TCR values of the MWCNT–Cu composites are unchanged up to 5 vol.% MWCNT, the TCR values of the MWCNT–CuCr composites significantly decreased with increasing MWCNT volume fraction and reached a value of 1451.1 × 10^–6^ °C^−1^ for the 5 vol.% MWCNT–CuCr composite. TCR of the composite is expected to decrease as the uniformly dispersed MWCNT/Cr_7_C_3_ hybrid filler increases. In the case of 10 vol.% MWCNT–CuCr composite fabricated in this research, however, the MWCNTs were not completely separated from each other in the Cu grain boundaries, indicating the formation of MWCNT bundles as shown in Fig. S6. Measured electrical conductivity of the composite was also dramatically decreased with about 4.245 × 10^7^ S m^−1^ (73.18% IACS). This decrease in electrical conductivity is mainly originated by the agglomeration of MWCNTs in the Cu matrix. Agglomeration of MWCNTs also decreases the total contact surface area between the MWCNTs and the Cu matrix, which resulted in a reaction inhibition between Cr atoms and MWCNTs during the sintering process. Therefore, it is expected that the TCR can be lowered if a high vol.% MWCNT/Cr_7_C_3_–Cu composite with uniformly MWCNT dispersion is successfully fabricated by using fine copper powder.

Figure 4c shows the correlation between the electrical resistivity and the TCR of the various materials containing MWCNT–CuCr composites fabricated in this research. The temperature dependent resistivity (or conductivity) of the alloy materials is determined by scattering of an electron from two different types of scattering process, scattering due to impurities and due to thermal vibrations of the alloy materials. This can be expressed as follows,

3 $$\rho ={\rho }_{T}+{\rho }_{I}$$

where *ρ* is the effective resistivity, *ρ*_*T*_ is the resistivity due to scattering by thermal vibrations only, and *ρ*_*I*_ is the resistivity due to scattering of electrons by impurities only. *ρ*_*T*_ is proportional to temperatures over room temperature (${\rho }_{T}\propto T$); this situation mainly occurs in pure metals. However*, ρ*_*I*_ is not affected by the temperature variation, which is the main reason for the low TCR of the alloy metals. Therefore, commonly used resistor materials are binary alloy materials such as CuNi, CuMn, NiCr, etc. Because TCR is inversely proportional to the resistivity in the alloy materials, these metals exhibit extremely high resistivity, which restricts the size and thickness of resistors for ultra-low value realization. Furthermore, Cu electrodes, due to their relatively large resistance variation compared with that of the resistor materials, can greatly affect the entire TCR of a low ohm resistor.

On the other hand, the MWCNT–CuCr composites containing MWCNT/carbide have superior properties of low electrical resistivity and low TCR, which it was not possible to attain simultaneously in conventional materials (Fig. 4c). The TCR of the CNT is deeply related to the wall thickness and diameter of the CNTs due to changes in the conducting routes. Naeemi and Meindl reported that TCR of MWCNTs decreases with decreasing CNT length and increasing CNT diameter; these values are calculated using an equivalent circuit model^21^. In the case of 50 nm diameter of MWCNT, TCR varies from about −2,500 ppm (corresponding to a 100 nm CNT length) to 4,000 ppm °C^−1^ (corresponding to a 1000 μm CNT length). Considering the diameter and length of the MWCNTs (*D*_MWCNT_: 20–110 nm, *L*_MWCNT_: 2–15 μm) used in this research, TCR of the MWCNTs will be much lower than −2,500 ppm °C^−1^ at room temperature. For MWCNTs with length shorter than roughly 14.5 μm, furthermore, an increasing temperature decreases the TCR of the MWCNTs.

*In-situ* formation of MWCNT/Cr_7_C_3_ hybrid structures by diffusion of Cr atoms to amorphous carbon and to defects of the MWCNTs decreases the interfacial resistivity of the composite.

When the carbide particles are simply mixed with MWCNTs and Cu powders, however, it is difficult to uniformly disperse the fine carbide particles between the MWCNT and Cu matrix. Furthermore, unstable interfacial properties between MWCNT/Cr_7_C_3_ and Cr_7_C_3_/Cu would deteriorate electrical properties of the composite. Consequently, separately dispersed or agglomerated carbide particles not only decrease electrical conductivity, but also aggravate TCR of the composite. In the case of MWCNT/Cr_7_C_3_–Cu composite system, however, Cr_7_C_3_ formed selectively on the MWCNT/Cu interface plays a role in deriving MWCNT’s excellent electrical conductivity and TCR properties. Consequently, MWCNT/Cr_7_C_3_–Cu structure possesses low TCR values without severe deteriorating of electrical conductivities compared with Cu, which has high TCR value of about 4,000 ppm °C^−1^.

## Conclusions

In summary, a Cu matrix composite having incompatible properties of both high electrical conductivity and low TCR has been successfully fabricated by using an SPS process to fabricate a thick and short multi-walled carbon nanotube (MWCNT)/Cr_7_C_3_ hybrid structure. The Cr_7_C_3_ nanostructures at the MWCNT/Cu interface, epitaxially grown by a reaction of diffused Cr atoms and amorphous carbon of the MWCNTs, can assist in improving the electrical properties of the MWCNT–CuCr composites by reducing amorphous carbons and defects of the MWCNTs. The MWCNT–CuCr composites, having high electrical conductivity and low TCR, are believed to be applicable as low Ω resistors or precise conducting materials in electronic components.

## Methods

### MWCNT–CuCr composite fabrication

CuCr (0.085 at.%) solid solution powder (Fukuda Metal Foil & Powder Co. Ltd., Japan), produced by gas atomization, was used in this study. Pristine MWCNTs (Hodogaya Chemical Co. Ltd., Japan) were used. The pristine MWCNTs were suspended in a 3:1 (v/v) mixture of concentrated sulfuric acid (H_2_SO_4_) (97%)/nitric acid (HNO_3_) (61%) and were ultrasonicated in a water bath at 50 °C for 24 h. The CuCr powder and treated MWCNTs were dispersed in an ethanol solution for 1 h by wet mixing with ultrasonication (Powersonic model 50, Yamato Scientific Company, Japan). These suspensions were carefully blended at different mixing ratios ranging from 0 to 5 vol.% MWCNTs and stirred for 30 min to generate heterogeneous suspensions. The mixtures were dried in an oven at 50 °C for 24 h. The fabricated MWCNT–CuCr mixed powders were consolidated by SPS (SPS-S515, SPS Syntex Inc., Japan). The MWCNT–CuCr mixed powders were then poured into a graphite die and sintered from 500 to 990 °C for 10 min by applying a uniaxial pressure of 50 MPa in vacuum. The heating rate was maintained at 50 °C min^−1^ up to the sintering temperature. The fabricated MWCNT–CuCr composites had a cylindrical shape with a diameter of 12 mm and a height of 10 mm.

### Measurements

The specimens were ground to 2400 grade using SiC papers, and were polished on a nylon cloth using diamond paste. All of the specimens were washed in an ultrasonic cleaner, rinsed with ethanol, and were finally dried in the oven. The bulk density of the composites was measured by the Archimedes method; values were then compared with the theoretical density to evaluate the relative density (RD). The theoretical density values of the CuCr powder and the MWCNTs were 8.95 and 2.1 g cm^−3^, respectively. The CuCr powder was embedded in an epoxy resin and hardened. To observe the microstructure, the samples were prepared by an ion milling system (GATAN PIPS, Model 691) before being examined by transmission electron microscopy (TEM) (HF-2000EDX, Hitachi, Japan). Microstructural characterization of the MWCNT–Cu composites was carried out by scanning electron microscopy (SEM) (JSM-6060, JEOL, Japan), field emission SEM (FESEM) (JSM-6500F, JEOL, Japan), and TEM.

Electrical resistivity of the composites was measured using a Van der Pawn method (ResiTest 8300, Toyo Corporation, Japan) at room temperature. Temperature dependent resistance of the composites was also measured using a 4-terminal measurement technique with an ultra-low resistance digital resistance checker (AX-1152D, ADEX, Japan) from room temperature to 125 °C in air. In order to obtain reliable results five measurements were conducted on each sample and the mean value of electrical conductivities and TCRs was used for further calculations throughout this paper. TCR values of the composites were calculated using the following equation:

$${\alpha }_{o}=\frac{1}{{\rho }_{o}}{[\frac{\delta \rho }{\delta T}]}_{T={T}_{o}}$$

where ${\alpha }_{o}$ is TCR (temperature coefficient of resistivity), $\delta \rho$ is the change in the resistivity, *ρ*_0_ is the resistivity at *T*_0_, $\delta T$ is the increase in temperature, and *T*_0_ is the reference temperature.

## Electronic supplementary material

Supplementary Information
