# Supplementary material for: Chromium carbide/Carbon Nanotube Hybrid Structure Assisted Copper Composites with Low Temperature Coefficient of Resistance
Source: Sci Rep. 2017 Nov 2;7:14943. doi: 10.1038/s41598-017-14915-7 (PMC5668293; doi:10.1038/s41598-017-14915-7)
Supplement: Supplementary file 1 — Supplementary Information [file 41598_2017_14915_MOESM1_ESM.docx]

**Chromium carbide/Carbon Nanotube Hybrid Structure Assisted Copper Composites with Low Temperature Coefficient of Resistance**

**Seungchan Cho^1,^*, Keiko Kikuchi2, Eunkyung Lee^3^, Moonhee Choi^4^, Ilguk Jo1, Sang-Bok Lee1, Sang-Kwan Lee1, and Akira Kawasaki2**

^1^Composites Research Division, Korea Institute of Materials Science (KIMS), Changwon 51508, South Korea

^2^Department of Materials Processing, Graduate School of Engineering, Tohoku University, Sendai 980-8579, Japan

^3^Mechanical Engineering, Worcester Polytechnic Institute, Worcester, MA01609, USA

^4^Samsung Electro-Mechanics, Suwon 16674, South Korea

**■ Supplementary Information for Publication**


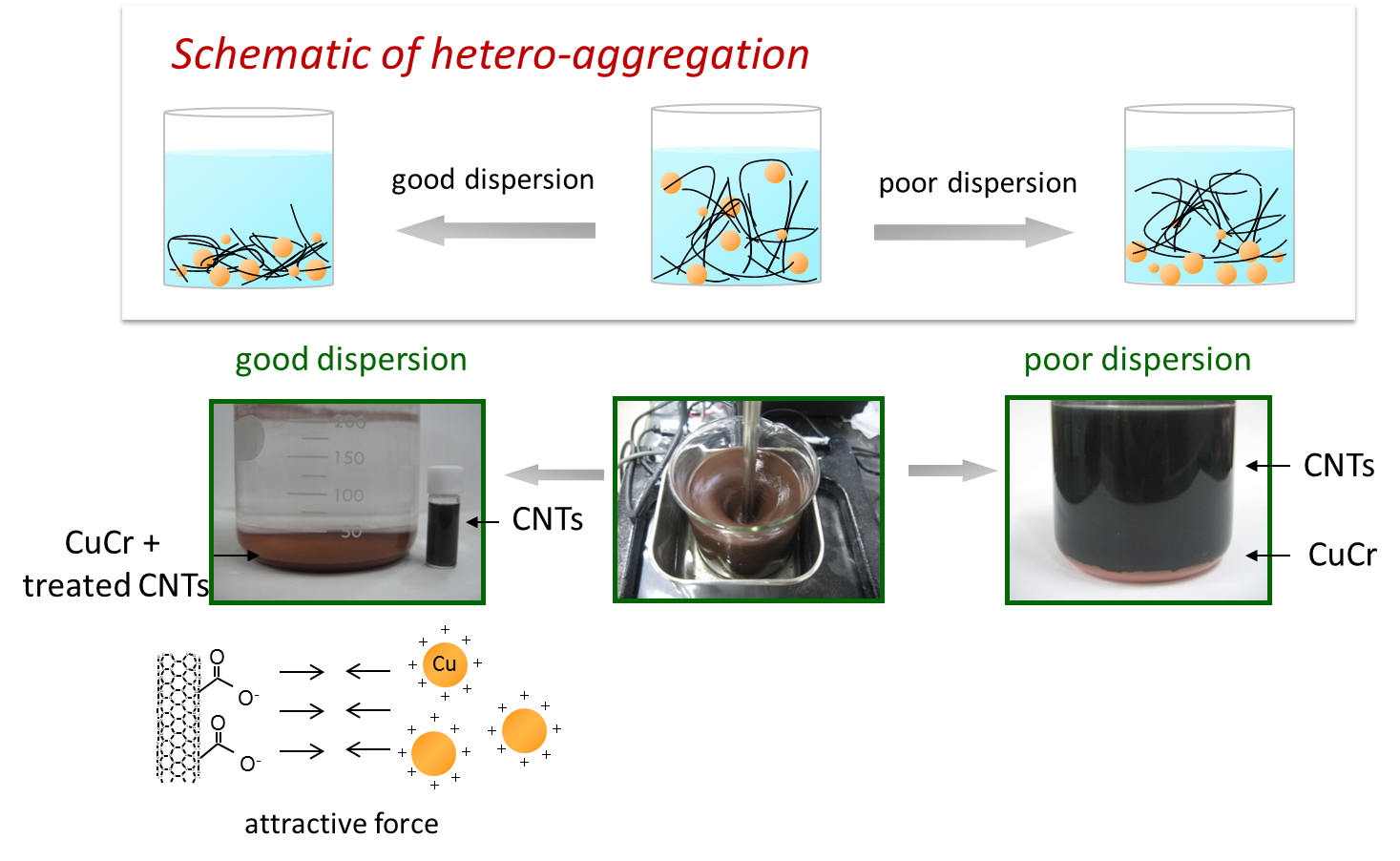


Figure S1. Schematic and experimental images of hetero-aggregation process of negatively charged MWCNTs and positively charged CuCr powders.


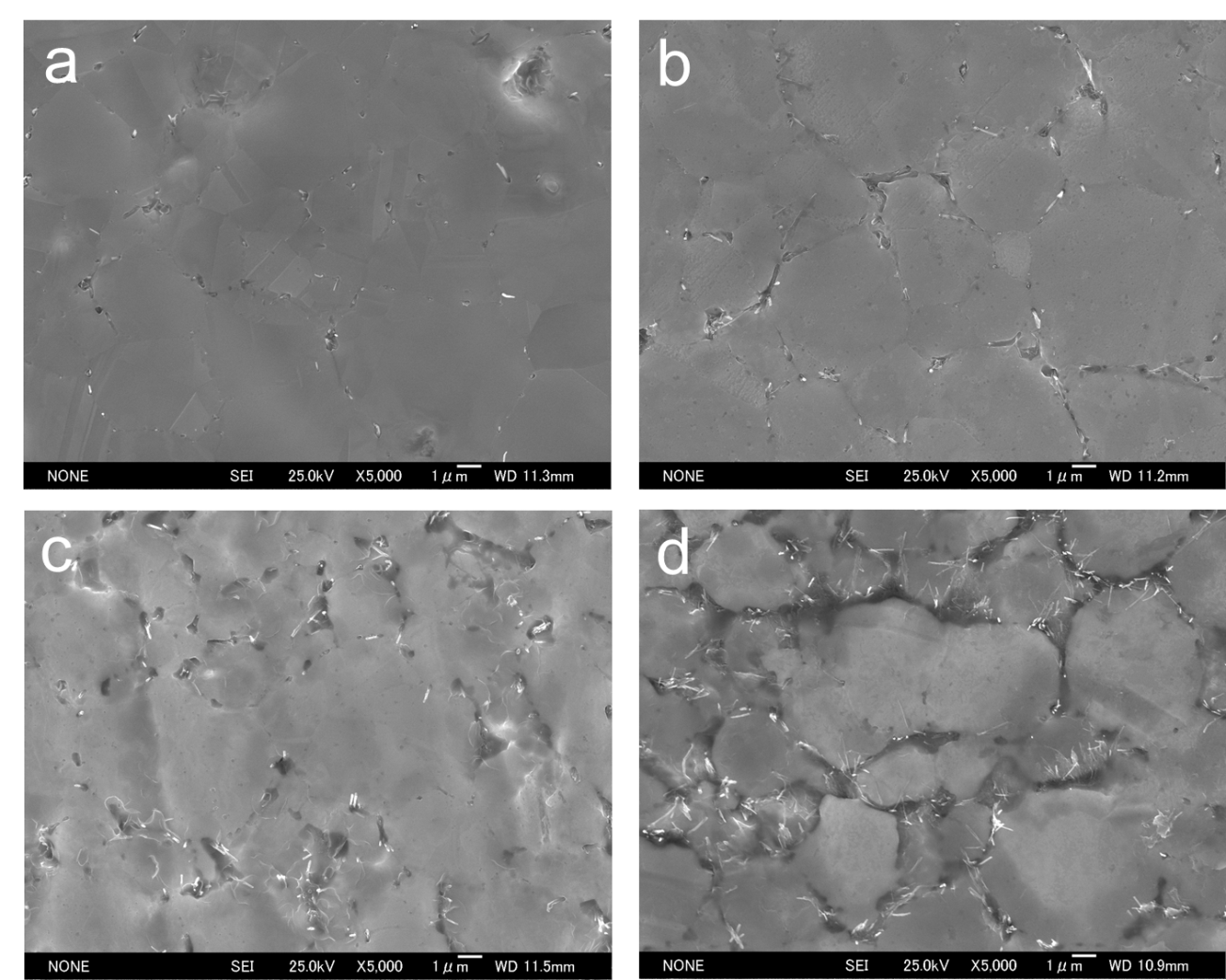


Figure S2. FESEM images of (a) 0.5 vol. %, (b) 1 vol. %, (c) 3 vol. %, and (d) 5 vol. % MWCNT–CuCr composites fabricated by SPS.


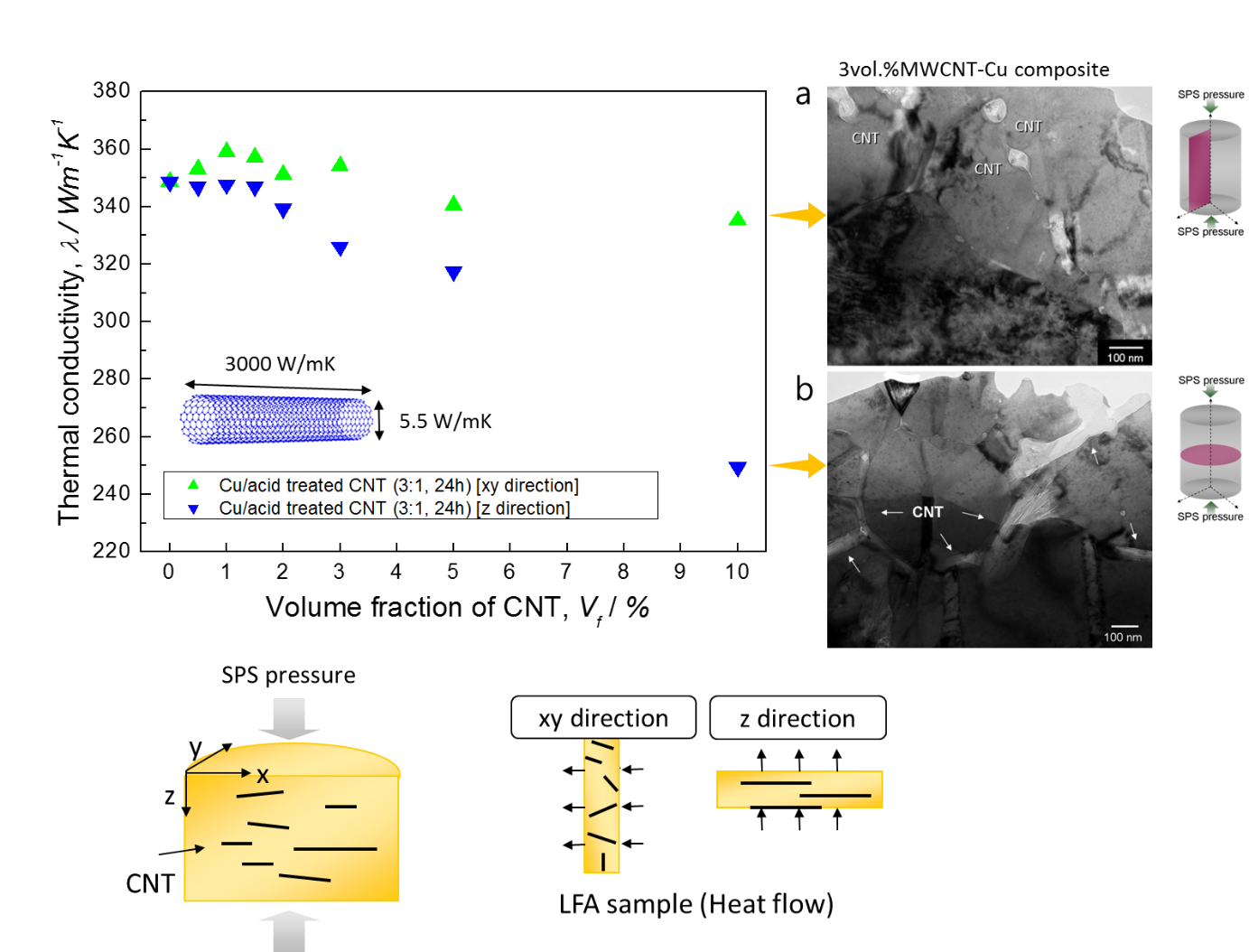


Figure S3. Thermal conductivities of MWCNT–Cu composites according to the measured direction, and TEM images in a plane (a) parallel, and (b) normal to the SPS compression axis of SPS indicating randomly oriented MWCNTs in a plane normal to the SPS compression axis.


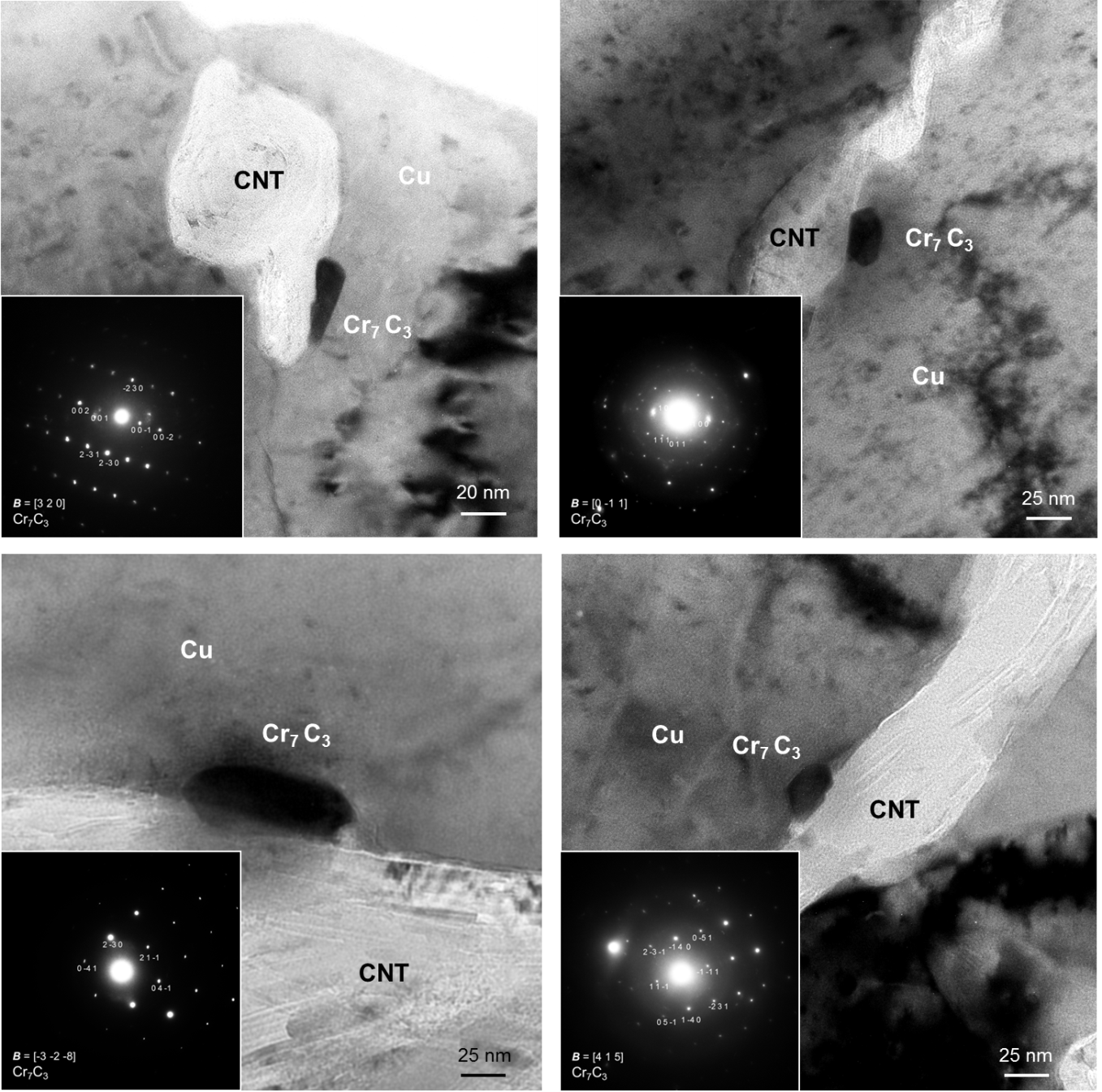


Figure S4. TEM images of MWCNT–CuCr composite and nano beam diffraction patterns of Cr carbide nanostructure generated at the surface of MWCNTs.


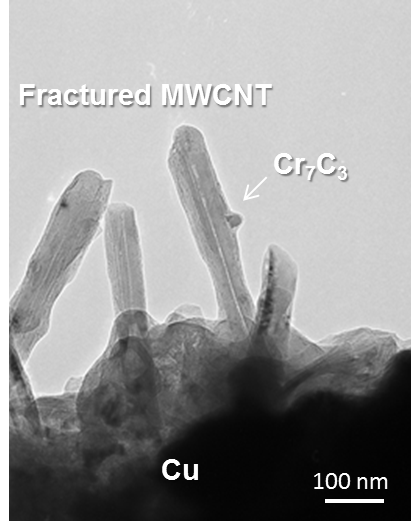


Figure S5. TEM image of fracture surface of MWCNT–CuCr composite after tensile test.


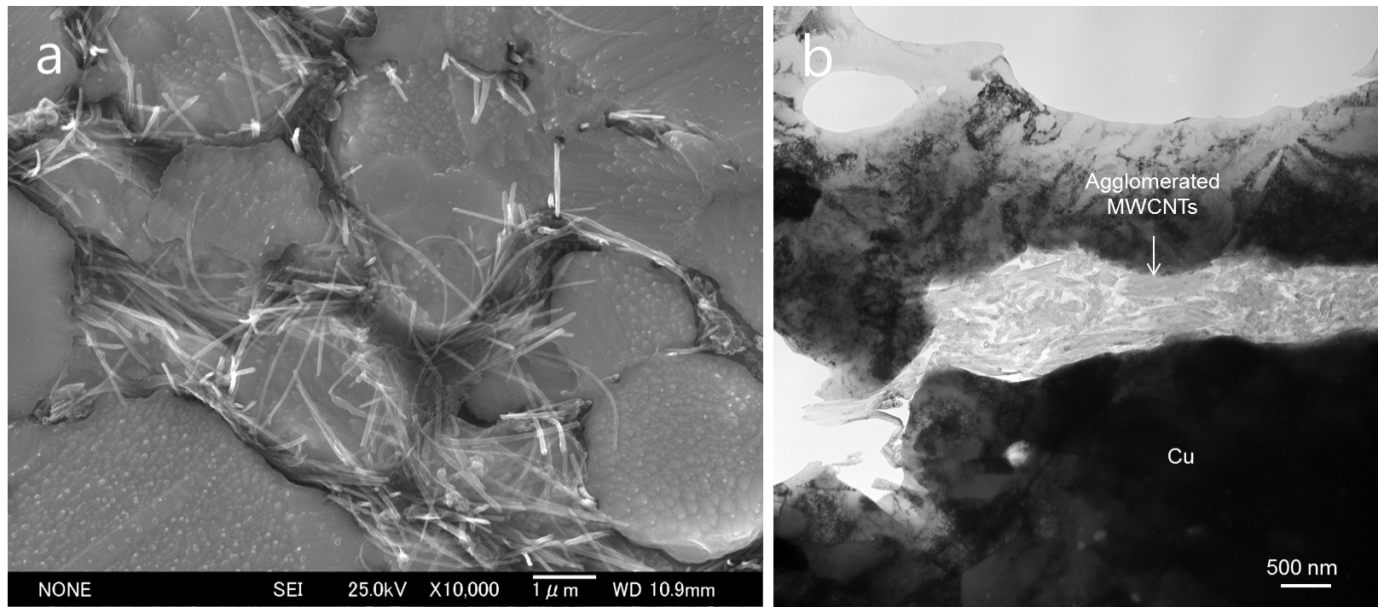


Figure S6. a) FESEM and b) TEM images of 10 vol.% MWCNT–CuCr composite indicating severe agglomeration of MWCNTs in the Cu matrix.
